# Vanadium Pentoxide-Based Composite Synthesized Using Microwave Water Plasma for Cathode Material in Rechargeable Magnesium Batteries

**DOI:** 10.3390/ma6104514

**Published:** 2013-10-11

**Authors:** Masashi Inamoto, Hideki Kurihara, Tatsuhiko Yajima

**Affiliations:** 1 Department of Applied Chemistry, Graduate School of Engineering, Saitama Institute of Technology, 1690, Fusaiji, Fukaya-shi, Saitama 369-0293, Japan; E-Mail: yajima@sit.ac.jp; 2 Saitama Industrial Technology Center, 3-12-18, Kamiaoki, Kawaguchi-shi, Saitama 333-0844, Japan; E-Mail: kuri@saitec.pref.saitama.jp

**Keywords:** microwave, rechargeable magnesium battery, cathode material, vanadium pentoxide, sulfur, manganese

## Abstract

Multivalent cation rechargeable batteries are expected to perform well as high-capacity storage devices. Rechargeable magnesium batteries have an advantage in terms of resource utilization and safety. Here, we report on sulfur-doped vanadium pentoxide (S-V_2_O_5_) as a potential material for the cathodes of such a battery; S-V_2_O_5_ showed a specific capacity of 300 mAh·g^−1^. S-V_2_O_5_ was prepared by a method using a low-temperature plasma generated by carbon felt and a 2.45 GHz microwave generator. This study investigates the ability of S-V_2_O_5_ to achieve high capacity when added to metal oxide. The highest recorded capacity (420 mAh·g^−1^) was reached with MnO_2_ added to composite SMn-V_2_O_5_, which has a higher proportion of included sulfur than found in S-V_2_O_5_. Results from transmission electron microscopy, energy-dispersive X-ray spectroscopy, Micro-Raman spectroscopy, and X-ray photoelectron spectroscopy show that the bulk of the SMn-V_2_O_5_ was the orthorhombic V_2_O_5_ structure; the surface was a xerogel-like V_2_O_5_ and a solid solution of MnO_2_ and sulfur.

## 1. Introduction

Recently, high-capacity rechargeable batteries have seen wide adoption as a power source for electric vehicles. Rechargeable magnesium batteries, which have been studied for a long time, have attracted attention for use in next-generation power storage applications. Aurbach *et al.* reported an electrolyte solution that allowed magnesium to dissolve and deposit reversibly [1,2].

However, there are a limited number of possible materials to use for the cathode of rechargeable magnesium batteries. In one case, Mg^2+^ is easily trapped in the cathode where it diffuses slowly. And, in another, repetitive insertion/desorption of Mg^2+^ at the cathode by use of high voltage can induce structural failure of the cathode or its dissolution into the electrolyte solution. Thus, one drawback of rechargeable magnesium batteries is the difficulty of maintaining their cycle characteristics because of their capacity fading [3,4].

For all of these reasons, there is high demand for cathode materials capable of stable insertion/desorption of Mg^2+^ in order to create a feasible rechargeable magnesium battery. The most commonly studied cathode materials are metal oxides [5,6] and metal sulfides [7,8]. As a rule, metal oxides possess stable crystal architecture but easily trap Mg^2+^. On the other hand, metal sulfides are less likely to trap Mg^2+^ because their structure is generally unsound. Since sulfides have generally rather lower bond-energy than oxides, those are thought to be unsuitable for the cathode material for lithium-ion batteries. Our research is advancing toward a solution to the above problem. A summary of our results is given below. 

**Figure 1:**
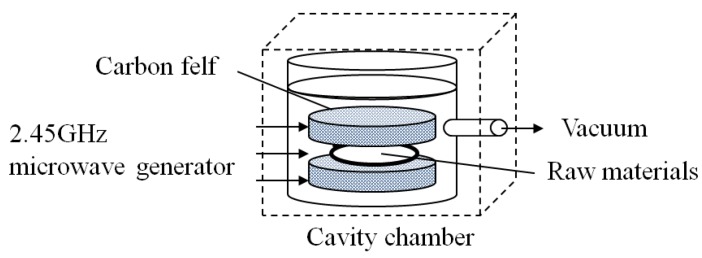
Schematic diagram of carbon felt and a 2.45 GHz microwave generator (CF-MWP).

Sulfur-doped vanadium pentoxide (S-V_2_O_5_) prepared by the method described in Section 3.1 was tested as an adaptation for the cathode of a rechargeable magnesium battery [9]. Our method employs low-temperature plasma generated using carbon felt and a 2.45 GHz microwave generator (CF-MWP, Figure 1) [10]. Structural analysis and charge-discharge characteristic evaluation were performed for S-V_2_O_5_ prepared by CF-MWP. 

**Figure 2:**
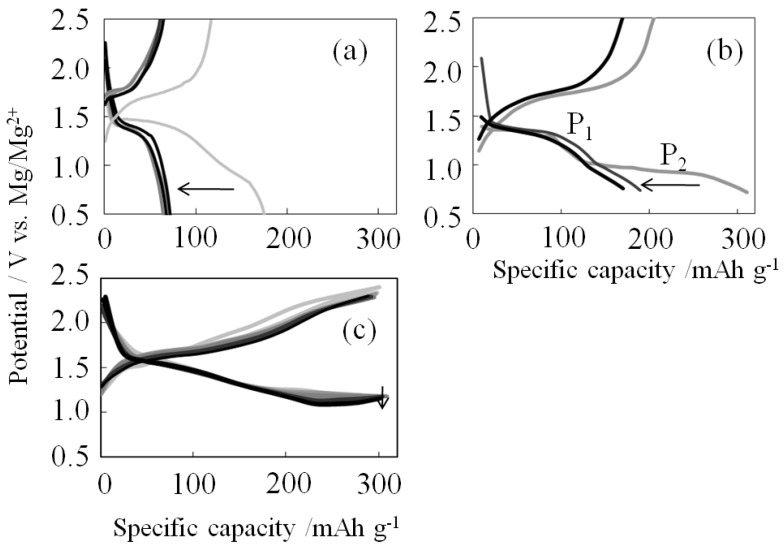
Charge-discharge curves: (**a**) V_2_O_5_; (**b**) Mixture of S and V_2_O_5_; and (**c**) S-V_2_O_5_.

Charge-discharge capacity curves are shown in Figure 2. For the orthorhombic V_2_O_5_ (Figure 2a), the first-cycle discharge was 170 mAh g^−1^, and the second-cycle discharge was 70 mAh g^−1^. This result is similar to that reported by Yu *et al.* [11]. For the composite of V_2_O_5_ and sulfur (Figure 2b) formed by simple mixing, two plateau potentials (P_1_ and P_2_) appeared at 1.4 V and 1.0 V *versus* Mg/Mg^2+^ due to V_2_O_5_ and sulfur, respectively. P_2_ appeared in the first discharge cycle but did not appear after the second discharge cycle. One possible reason is that the sulfur may have been transferred to the electrolyte solution at the first Mg^2+^ desorption. However, the discharge capacity curve of S-V_2_O_5_ (Figure 2c) was 300 mAh·g^−1^ and decreased in a linear fashion from 1.6 V to 1.0 V *versus* Mg/Mg^2+^. This result implies that the surface of S-V_2_O_5_ became amorphous [12]. 

**Figure 3:**
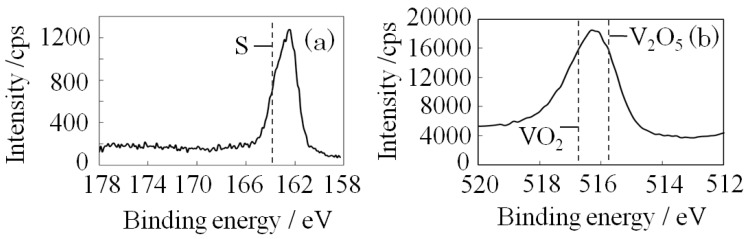
X-ray photoelectron spectroscopy (XPS) narrow spectra of S-V_2_O_5_: (**a**) S2p; and (**b**) V2p_3/2_.

The X-ray photoelectron spectroscopy (XPS) results for S-V_2_O_5_ (Figure 3) showed that the S2p spectrum shift occurred at low energy and the V2p_3/2_ spectrum shift occurred at a high energy. These results indicate a V-S bond-like state at the surface of S-V_2_O_5_ because of the solid solution formation of sulfur and V_2_O_5_. Results of FTIR, XRD, SEM, and EPMA testing indicate that the surface of S-V_2_O_5_ was amorphous, similar to a xerogel structure, and that the bulk was the orthorhombic V_2_O_5_. 

The electrolyte after a charge-discharge cycle of S-V_2_O_5_ electrode included sulfur, which it did not include originally. This suggests that sulfur leeched from the S-V_2_O_5_ electrode into the electrolyte through Mg^2+^ intercalation and desorption. Therefore, this study discusses the addition of a metal oxide material to inhibit sulfur elution. 

## 2. Results and Discussion

### 2.1. Electrochemical Characteristics

**Figure 4:**
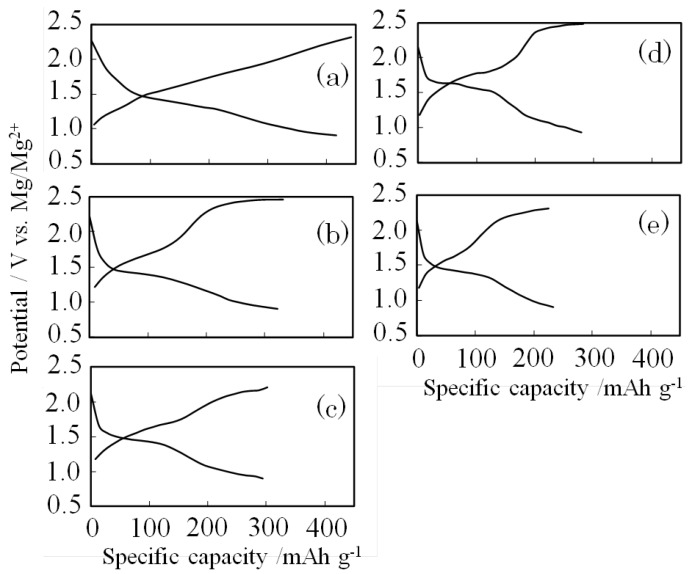
Charge-discharge curves at second cycle: (**a**) MnO_2_; (**b**) MoO_3_; (**c**) Fe_2_O_3_; (**d**) NiO; and (**e**) ZrO_2_.

Charge-discharge capacity curves are shown in Figure 4. The discharge capacities of the batteries containing metal oxides (MnO_2_, MoO_3_, Fe_2_O_3_, NiO, and ZrO_2_) as additives were 420 mAh g^−1^, 320 mAh g^−1^, 300 mAh g^−1^, 290 mAh g^−1^, and 230 mAh g^−1^, respectively. The highest capacity was reached in the case of MnO_2_ (SMn-V_2_O_5_). The discharge curve in the case of SMn-V_2_O_5_ decreased linearly from 1.5 V to 0.9 V *versus* Mg/Mg^2+^. This result demonstrates that the surface of the SMn-V_2_O_5_ was amorphous, similar to a xerogel structure; in this regard, it is similar to S-V_2_O_5_. Although the discharge curve obtained with added MoO_3_ decreased linearly from 1.5 V to 0.9 V *versus* Mg/Mg^2+^, its charge curve showed plateau potentials at around 1.8 V and 2.4 V. In the other curves, a plateau potential appeared at 1.5V, and each of the curves descended abruptly after the plateau potential. The cathode formed by the addition of NiO had the highest plateau potential at 1.65 V, different from other cases. This result shows that the addition of NiO might improve the high-voltage characteristics of the obtained material. The metal oxides other than MnO_2_ inhibited the formation of an S-V_2_O_5_ amorphous structure and showed a plateau potential. 

### 2.2. Structural Analysis 

**Figure 5:**
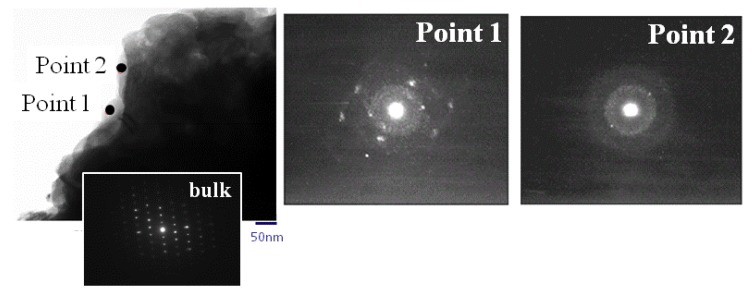
Transmission electron microscopy (TEM) of S-Mn-V_2_O_5_ and electron beam diffraction at Points 1 and 2.

**Figure 6:**
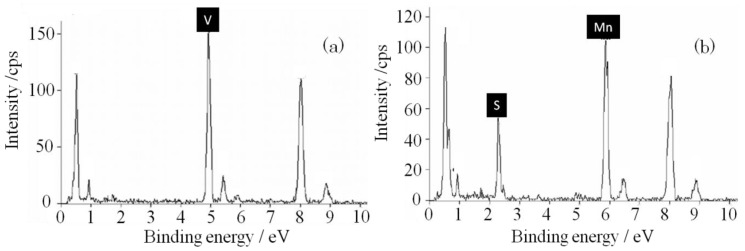
Energy dispersive X-ray spectrometry (EDX) at (**a**) Point 1; and (**b**) Point 2.

A transmission electron microscopy (TEM) image, electron beam diffraction image, and energy dispersive X-ray spectrometry (EDX) spectrum of SMn-V_2_O_5_ are shown in Figure 5 and Figure 6. The bulk of the SMn-V_2_O_5_ produced a clear image of electron diffraction. This result corresponds with the pattern of the orthorhombic V_2_O_5_. Therefore, the bulk of the SMn-V_2_O_5_ has maintained the V_2_O_5_ the orthorhombic structure without degradation. In the TEM image, the surface of SMn-V_2_O_5_ is seen as a thin layer with two types of morphology, shown in Figure 5 as Points 1 and 2. The diffraction image of Point 1 has a clear diffraction pattern and halo pattern. This diffraction pattern is slightly broader than the pattern for the orthorhombic V_2_O_5_, and the observation of the halo pattern indicates an amorphous structure. The EDX spectrum at Point 1 shows a strong V peak. These results indicate that Point 1 was a V_2_O_5_ xerogel or a similar structure. This is similar to the case of S-V_2_O_5_. The diffraction image of Point 2 shows only a halo pattern, and the EDX spectrum shows peaks for manganese and sulfur. Because these results indicate that Point 2 has an amorphous structure and consists of only manganese and sulfur, it is likely that Point 2 is a solid solution of MnO_2_ and sulfur. 

**Figure 7:**
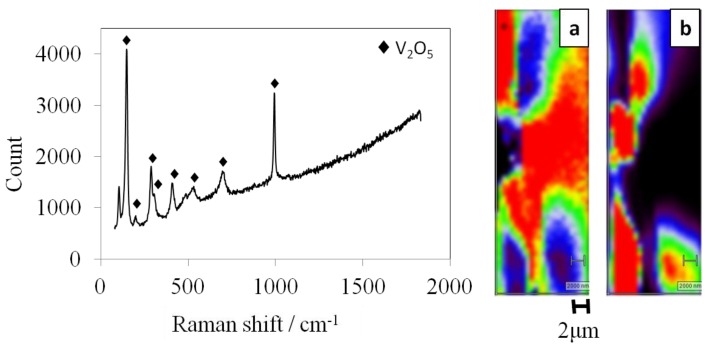
Raman spectroscopy of SMn-V_2_O_5_ and Raman images of the surface of the SMn-V_2_O_5_ of (**a**) V_2_O_5_; and (**b**) the fluorescence area.

The micro-Raman spectrum of an SMn-V_2_O_5_ electrode is shown in Figure 7. The orthorhombic V_2_O_5_ peaks [13] and fluorescence can be seen, whereas there are no MnO_2_ and sulfur peaks. This result indicates fluorescence, possibly derived from MnO_2_ and sulfur. Thus, during Raman spectroscopy, although the area of fluorescence was low in intensity for V_2_O_5_, the orthorhombic V_2_O_5_ and the fluorescence area were separate. These results, together with the results from TEM and EDX tests, suggest that the fluorescence area was a solid solution of MnO_2_ and sulfur, indicating that SMn-V_2_O_5_ had a surface of the orthorhombic V_2_O_5_ covered with a solid solution. This is consistent with the TEM and EDX data. 

**Figure 8:**
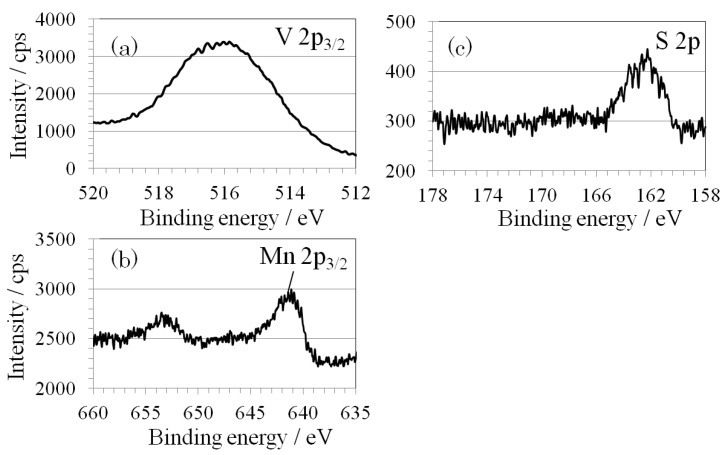
XPS narrow spectra of SMn-V_2_O_5_: (**a**) V2p_3/2_; (**b**) Mn2p_3/2_; and (**c**) S2p.

The XPS narrow spectrum of SMn-V_2_O_5_ is shown in Figure 8. The peaks for V2p_3/2_ and S2p at the same positions as S-V_2_O_5_ suggest a S-V bond-like state and indicate an amorphous structure. The FWHM of SMn-V_2_O_5_ increased 2-fold compared to S-V_2_O_5_. Therefore, there was considered that the oxidation state of V of SMn-V_2_O_5_ have slightly different compared to S-V_2_O_5_. Mn2p_3/2_ became a solid-solution formation with sulfur, according to another analysis. The ratio of manganese to sulfur was about 1:2. This ratio remained constant when the amount of sulfur was increased five-fold. These results demonstrate that MnO_2_ and sulfur were linked by mechanical force rather than by a chemical binding force. The ICP-MS analysis result shows that the molar ratio of V:Mn:S was 100:6.8:14.4. The sulfur content of SMn-V_2_O_5_ was twice that of S-V_2_O_5_ (V:S = 100:7.8). The ratio of manganese to sulfur was about 1:2 in SMn-V_2_O_5_; this result was arrived at by XPS analysis. 

Balanced chemical reactions involving V_2_O_5_, MnO_2_, and S, along with their theoretical capacities, are shown below.

V_2_O_5_ + Mg^2+^ + 2e^−^ → MgV_2_O_5_  294 mAh g^−1^ (1)

2MnO_2_ + Mg^2+^ + 2e^−^ → MgMn_2_O_4_  307 mAh g^−1^ (2)

S + Mg^2+^ + 2e^−^ → MgS     1674 mAh g^−1^ (3)

From the results of ICP-MS analysis and the three equations above, average theoretical capacities of SMn-V_2_O_5_ and S-V_2_O_5_ through the surface to bulk are calculated to be 458 mAh·g^−1^ and 393 mAh·g^−1^, respectively. That is, the empirically obtained capacity of SMn-V_2_O_5_ was 91.7% of the theoretical capacity. Because the theoretical capacity of MnO_2_ is as high as that of V_2_O_5_, achieving a high capacity for SMn-V_2_O_5_ may be possible by increasing the amount of sulfur.

## 3. Experimental Section 

### 3.1. Preparation of Cathode Material by CF-MWP

Vanadium pentoxide (V_2_O_5_), sulfur, and a metal oxide (MnO_2_, MoO_3_, Fe_2_O_3_, NiO, or ZrO_2_) were mixed at a molar ratio of 2:1:1 in a ball mill (P-6, Fritsch Co., Ltd.). Additionally, the composite added MnO_2_ was prepared the composite the ratio of 2:5:1 because of observing increase of sulfur contained amount. This composite was wetted down and left overnight, after which it was treated by our method involving low-temperature plasma generated using carbon felt and a 2.45 GHz microwave generator (CF-MWP). Specifically, 2.0 g of each raw material were placed between pieces of carbon felt (30 mm in diameter) and a 500 W, 2.45 GHz microwave was used to irradiate the material under reduced pressure (0.001 MPa) for 2 min (Figure 1) to synthesize the hybrid cathode materials. In this process, raw materials are treated by plasma formed from water in the raw materials as a result of electric discharge between the pieces of carbon felt. It is assumed that the water in the raw materials is distributed uniformly enough that the composite is treated uniformly. Furthermore, although the process was performed under reduced pressure and at the evaporating temperature of water, the process did not induce reduction of V_2_O_5_ and oxidation of sulfur. 

### 3.2. Electrochemical Characteristics

The electrodes were prepared from a mixture of the cathode material, acetylene black, and a polyvinylidene fluoride binder with *N*-methyl-2-pyrrolidone with a weight ratio of 10:3:1. The resulting slurry was spread on carbon paper. The electrode was dried at 110 °C for 1.5 h. S-V_2_O_5_ was charged with magnesium ions and used as a counter electrode. This electrode showed the same potential changes as a magnesium alloy plate. A magnesium alloy plate was used as the reference electrode. For the electrolytic solution, 0.3 M Mg(ClO_4_)_2_ and 1.8 M H_2_O dissolved in propylene carbonate were used, and the electrode performance was evaluated using three-electrode cells. The electrolyte has been proven for metal oxides as cathode active materials to charge and discharge smoothly [4]. The stability of the Mg pseudo reference electrode used was preliminarily checked to be stable by observing the reproducibility of cyclic voltammograms of ferrocene using the electrolytic solution containing 0.2 M ferrocene, 0.3 M Mg(ClO_4_)_2_ and 1.8 M H_2_O in propylene carbonate solvent and an electrochemical system with a magnesium alloy plate as the reference electrode and platinum wires as working and counter electrodes. Charge-discharge tests were conducted between cut-off potentials from 2.4 V to 0.9 V *versus* Mg/Mg^2+^ at a constant current of 60 mA·g^−1^ (0.1 C). All trials were conducted at 25 °C. 

### 3.3. Structural Analysis 

The as-prepared samples were characterized by TEM (HF2000, Hitachi, Japan), EDX (Noran System SIX, Thermo Fisher Scientific, USA), micro-Raman spectroscopy (in Via Reflex/StreamLine, Renishaw, UK), and XPS (Quantum 2000, PHI, Inc.). The Raman spectroscopy measurements were taken after the electrodes were prepared. 

## 4. Conclusions

As a cathode material for rechargeable magnesium batteries, we synthesized S-V_2_O_5_ with an added metal oxide prepared by CF-MWP and examined its crystal structure and electrode characteristics. The composite of V_2_O_5_, sulfur, and MnO_2_ (SMn-V_2_O_5_) synthesized by CF-MWP demonstrated the highest capacity of 420 mAh·g^−1^ of any of the prepared samples. The charge-discharge curves showed that the SMn-V_2_O_5_ capacity decreased linearly from 1.5 V to 0.9 V but that, for the other electrodes, a plateau potential appeared at 1.5 V. This result was interpreted to mean that only SMn-V_2_O_5_ had a surface structure resembling a xerogel. The composite of V_2_O_5_, sulfur, and MnO_2_ was treated by CF-MWP. The bulk of the resulting composite was orthorhombic V_2_O_5_, while the surface showed a xerogel-like structure of V_2_O_5_ and a solid solution of sulfur and MnO_2_. 
